# Microbial Eukaryotes: Extending Observations North

**DOI:** 10.3389/fmicb.2013.00010

**Published:** 2013-02-05

**Authors:** Jennifer F. Biddle

**Affiliations:** ^1^College of Earth, Ocean and Environment, University of Delaware, Lewes, DE, USA

Free-living microbial eukaryotes are an enigmatic group of organisms. Many have been discovered through traditional cultivation and microscopic observation, with continued observations leading to the acknowledgment that many small eukaryotic species are potentially dispersed at a global level (Finlay, 2002). While cell size could affect global distribution, habitat selection could allow for abundances of organisms to show environmental preferences, making widespread investigations of microbial eukaryotes through multiple analysis methods valuable for understanding these unseen species.

In recent years, investigations into the diversity of microbial eukaryotes have revealed a massive diversity that had been previously overlooked. While knowledge has been gained in more accessible environments, extreme environments that may show drastic habitat preference for microbial eukaryotes have been less sampled. Within pelagic environments, mixing was thought to lead to a more even distribution of organisms, however, the empirical evidence of habitat selection has often led to the concept of moderate endemicity where only a few species are cosmopolitan, and others are endemic (Foissner, 2006). In polar environments, water masses have been shown to separate bacteria into biomes (Galand et al., 2010) and the Arctic and Southern Ocean have been shown to be distinguishable from each other (Ghiglione et al., 2012), suggesting physical factors exert large controls that overcome dispersal and allow habitat specificity of small cells.

In polar environments, water, sea ice, and snow represent biomes much like polar water masses, that are physically interconnected yet separate, with some organisms from water freezing into and surviving within sea ice and snow being physically connected but potentially seeded with organisms from the atmosphere. Eukaryotes examined in sea ice have mostly been phototrophic species, with diatoms the most frequently examined group (Staley and Gosink, 1999) although other groups have been detected (Brown and Bowman, 2001). The idea of habitat selection of microbial eukaryotes has been hampered in this environment through methodological differences in approaches, with microscopic investigations showing good overlap of the ice and water biomes, but molecular analyses showing habitat specificity (Mock and Thomas, 2005). As such, polar environments hold a key to understanding the global footprint of microbial eukaryotes and their potential barriers to dispersion. The sea ice biome is also recognized to be active (Wheeler et al., 1996) and subject to change (Kirchman et al., 2009; Montes-Hugo et al., 2009), making knowledge about the current polar water, sea ice, and snow diversity valuable for baseline values.

Recently a study by Bachy et al. (2011) has extended the knowledge on microbial eukaryotes to the region near the North Pole. This first investigation into the Arctic offshore environment allows a glimpse at a complex ecosystem that includes multiple biomes of snow, sea ice, and water. The study took place on a large floe of ice that freely drifted during the field season, and samples were taken at the end of the polar night, when the ice had drifted south of the exact North Pole. The permanent ice cover of this environment allowed for a vertical profile of snow, multi-year ice, and underlying water to 170 m below sea level to be examined using 18S rDNA cloning and sequencing, allowing for thorough investigation into the microbial eukaryote community. Additionally, the study comes at the end of the polar night, where phototrophic species are drastically reduced.

The study found that seawater held by far the largest microbial eukaryote diversity, followed by sea ice and distantly by snow. The diversity of operational taxonomic units (OTUs) in the deep ice in contact with the upper water was extremely similar, yet the taxon abundances were dissimilar, with shallow water holding more Fungi than the deepest ice. Diversity increased through the ice, with the most recent years of shallow ice containing lower diversity than deeper ice, suggesting that inputs are different between layers, or organisms are showing adaptation to the habitat over time. Detailed analysis of the sequences show that some species could be interpreted as originating in the water column and entering the deep overlying ice, likely during freezing, suggesting that the deep ice more closely resembles the community in the shallow waters. Snow had low diversity and the sequences detected in it are suggestive of potential contaminants, including humans and temperate plants. As such, eukaryotic sequences, such as those associated with pollen from *Pinus* species, may represent dispersed, but inactive eukaryotes unaffected by habitat selection.

Activity is inferred in this study from the lack of phototrophs detected. Typically, abundant diatom species are observed in ice and water samples, however, at the end of the polar night, the light starved community had decreased to lower levels. Many of the detected organisms were related to known mixotrophs and diatom predators were detected, suggesting that the primary production within the community was low and impacted by grazing (Bachy et al., 2011). These results reveal the resilience of mixotrophs, enduring 6 months of darkness. Work performed seasonally in the Antarctic agrees with this community variability, showing that summer and winter analyses show different bacterioplankton communities (Grzymski et al., 2012). These studies suggest that polar communities are fluctuating on a drastic seasonal basis, despite their recognized low activities (Wheeler et al., 1996). Future climate change may shift these population changes, so baseline levels during winters are valuable (Schofield et al., 2010).

The glimpse by Bachy et al. (2011) into the polar community of the high Arctic Ocean shows unique features of a stratified microbial eukaryote community, separated by physical boundaries. The comparison of habitats suggests that populations of mixotrophs may be rare in seasonal situations, then bloom when their comparative fitness is increased by the lack of light. As such, deep sequencing surveys of eukaryotic populations may assist with the unraveling of the mysteries of global microbial eukaryote dispersal. Continued investigations into these difficult to reach polar environments will allow for full global analysis to be considered when determining cosmopolitan microbial eukaryote species and how their abundance and activity are affected in a changing climate.
